# Study of the Properties of a Biodegradable Polymer Filled with Different Wood Flour Particles

**DOI:** 10.3390/polym12122974

**Published:** 2020-12-13

**Authors:** Francisco Parres, Miguel Angel Peydro, David Juarez, Marina P. Arrieta, Miguel Aldas

**Affiliations:** 1 Departamento de Ingeniería Mecánica y Materiales, Universitat Politècnica de València, Plz Ferrándiz y Carbonell, s/n, 03801 Alcoy, Spain; mpeydro@mcm.upv.es (M.A.P.); djuarez@mcm.upv.es (D.J.); 2 Departamento de Ingeniería Química y del Medio Ambiente, Escuela Politécnica Superior de Ingenieros Industriales, Universidad Politécnica de Madrid (ETSII-UPM), Calle José Gutierrez Abascal 2, 28006 Madrid, Spain; m.arrieta@upm.com; 3 Grupo de Investigación, Polímeros, Caracterización y Aplicaciones (POLCA), 28006 Madrid, Spain; 4 Departamento de Ciencia de Alimentos y Biotecnología, Facultad de Ingeniería Química y Agroindustria, Escuela Politécnica Nacional, Quito 170517, Ecuador; miguel.aldas@epn.edu.ec

**Keywords:** biopolymer, Solanyl^®^, wood flour, Lignocel^®^, lignocellulosic particles, fuller method, mechanical properties, rheological characterization

## Abstract

Lignocellulosic wood flour particles with three different sizes were used to reinforce Solanyl^®^ type bioplastic in three compositions (10, 20, and 30 wt.%) and further processed by melt-extrusion and injection molding to simulate industrial conditions. The wood flour particles were morphologically and granulometric analyzed to evaluate their use as reinforcing filler. The Fuller method on wood flour particles was successfully applied and the obtained results were subsequently corroborated by the mechanical characterization. The rheological studies allowed observing how the viscosity was affected by the addition of wood flour and to recover information about the processing conditions of the biocomposites. Results suggest that all particles can be employed in extrusion processes (shear rate less than 1000 s^−1^). However, under injection molding conditions, biocomposites with high percentages of wood flour or excessively large particles may cause an increase in defective injected-parts due to obstruction of the gate in the mold. From a processing point of view and based on the biocomposites performance, the best combination resulted in Solanyl^®^ type biopolymer reinforced with wood flour particles loaded up to 20 wt.% of small and medium particles size. The obtained biocomposites are of interest for injected molding parts for several industrial applications.

## 1. Introduction

The consumption of polymers has been steadily increasing since they first appeared. It would be difficult to imagine life today without these materials. Thus, the world plastic production is nowadays about 350 million tons per year [1]. The most significant problem with traditional polymers is the amount of plastic waste, particularly those coming from short-term applications such as packaging. Although a high amount of plastic wastes is recycled every year, there still is a great problem regarding the large number of plastic wastes that finishes in landfills.

Despite the existence of photodegradable and biodegradable polymers, the design of many landfills is not efficient [2]. For this reason, recycling, reuse, composting, and incineration seem to be the most environmentally and feasible way to reduce plastic waste after their useful life. There is a special scientific interest in the use of more sustainable polymers in the packaging field (i.e., biopolymers and/or reusable packaging) to reduce the disposal of plastic waste in landfills. Moreover, it should be considered that landfills should be adapted to current plastic wastes [3,4]. Biopolyesters have emerged as the most promising sustainable polymers to replace traditional thermoplastics used for packaging materials, mainly due to their easy processability because of their available processing technologies at the industrial level (i.e., melt blending, extrusion, injection molding, etc.) [5,6]. Additionally, great efforts have been made in rheological [7] and mechanical properties [3] research in biodegradable polymers. These properties are essential for plastic processing manufacturing and their further application in the industrial sector.

Solanyl^®^ is a bio-based material based on reclaimed potato starch [8], the main component of which is the poly(lactic acid) (PLA), as is further confirmed by the DSC technique in the results section of this work. PLA is nowadays, undoubtedly, the most attractive biodegradable polymer for rigid and flexible packaging applications, due to its many advantages such as availability in the market, ease of processing, economic competitiveness, high transparency, and environmentally friendly characteristics [5,9]. However, PLA possesses some disadvantages such as poor thermal, mechanical, and barrier properties [10,11]. Thus, considerable academic and industrial efforts have been focused on improving PLA performance, such as copolymerization or blending with others biopolyesters such as poly(ε-caprolactone) (PCL) and/or poly(hydroxy alkanoates) (PHAs) [12,13,14], mainly focused on increasing its crystallinity for extending the industrial applications. Although many PLA-based biodegradable polymers are currently commercialized, they are not widely used at the industrial level, mainly due to the substantially higher price of biopolymers compared to the more conventional petroleum-derived and/or non-degradable polymers. On the other hand, there are many ways to reduce the price of a material. First, and perhaps most difficult, is the use of cheaper raw materials. Second, there is the possibility of generating a more widespread use of material, in which case demand and production would increase, while costs would naturally decrease. A third option is to introduce a reinforcing material or fillers into the polymeric matrix by developing composites and/or nanocomposites [15]. This approach is probably the most currently scalable option in the industrial sector. Polymeric composites have been used to reduce the cost of producing plastic materials. However, to guarantee the green character of the final polymeric formulation, the filler materials should be also biobased and/or biodegradable. Thus, during the last decades, many research works have been focused on the development of biocomposites and bionanocomposites, for improving the different properties of the materials.

In this context, several authors have analyzed the effect of different particles (i.e., chitosan, cellulose nanocrystal [15,16], clay and silver nanoparticles, organomontmorillonite/graphene [17], kraft lignin [18] and TiO_2_ [19], among others, on different properties of biopolymers [20,21,22,23]. Additionally, others have analyzed the degradation process of biopolymers in detail using Taylor Dispersion Analysis (TDA) [24]. Nowadays, the use of residues as raw material sources for lignocellulosic derivatives production is considered to be one of the most promising strategies for reducing industrial waste [25]. Lignocellulose derivatives can be obtained from agricultural by-products of several industries [26,27]. They seem to be optimal reinforcing fillers for biopolymeric matrices since they are biobased, biodegradable, lightweight, stiff, non-abrasiveness to the processing equipment, and highly abundant in nature at low cost [15,16]. Although nanocellulose has been recognized in recent years as one of the most interesting reinforcement for biopolymers due to its extraordinary performance [15,16,25,27], the extraction process from agricultural sources requires bleaching treatments, basic treatments, and further the isolation of cellulose nanocrystals from cellulose fibers by acid hydrolysis [27]. Thus, nanocellulose production at an industrial scale is still in development, and it is not cost-effective for the plastic processing industry. In this regard, the wood industry produces a wide range of by-products which can be used to gain value as derivatives in many fields, for example, bark on biofuel [28], in agricultural fields as an alimentary complement for cows [29], animal bedding, for wood-smoking meat; and in materials field as an absorbent material and as reinforcing fillers for the development of composites. Wood flour has been studied and analyzed as a filler in different materials. It is common to use coupling agents to improve interactions between particles and polymers, these results show improving the adhesion between particle and matrix, and thermal stability [30,31].

On this basis, the present work aims to study the influence of wood flour particle size, at the microscale level, on the properties of biodegradable composite materials based on Solanyl^®^ type bioplastic. Wood flour with different granulometry, ranging from 70 to 1100 µm, was assessed as filler. Commercial wood flour particles with three different sizes were selected: Lignocel^®^ CB120 (70–150 µm), Lignocel^®^ BK 40-90 (300–500 µm), and Lignocel^®^ Grade 9 (800–1100 µm). The lignocellulose particles were added at three different loadings (10, 20, and 30 wt.%) to reinforce the Solanyl^®^ type matrix. The materials were processed by melt-extrusion followed by injection molding processes to simulate the industrial processing conditions. The obtained formulations were assessed by rheological, mechanical, and thermal characterization to study the processability and performance of Solanyl^®^/Lignocel^®^ formulations, the influences of the size of lignocellulose particles as well as the suitability of these materials for industrial production in the plastic sector.

## 2. Materials and Methods

### 2.1. Materials

Solanyl^®^ C1201 (IMCD España Especialidades Quimicas, S.A., Barcelona, Spain) and three different wood flour particles, Lignocel^®^ CB120 (0.07–0.15 mm), Lignocel^®^ BK 40-90 (0.30–0.50 mm) and Lignocel^®^ Grade 9 (0.80–1.10 mm) (Rettenmaier Iberica S.L. y Cia. S. Com, Grupo JRS GmbH, Barcelona, Spain), were used in the study. The characteristics of the wood flour particles are reported in Table 1.

### 2.2. Granulometric Analysis

The particle size distribution of wood flour was determined by sieving. For this purpose, different sieves (2000 μm, 1000 μm, 500 μm, 250 μm, 125 μm, 63 μm, and bottom) were employed. The sieving machine used was CISA SIEVE SHAKER, model RP09 (Barcelona, Spain) with a sample of 200 g. The particle size distribution was calculated following the Fuller and Thompson method; Equation (1):

(1) y=100(dD)e

where:

*d* is the sieve size (in mm) being considered (32, 16, 8, 4, 2, 1, 0.5, 0.25, 0.125, 0.063). *d* value is always minor or equal to *D* value.
*D* is maximum particle size (in mm). *D* value is equal to size mesh with cumulative retained less to 15%
*e* is the parameter that adjusts the curve. *e* value is 0.5 to Fuller and Thompson method.

### 2.3. Optical Measurements

Morphological characteristics (length and diameter) of different wood flour particles were analyzed using a compact stereomicroscope OLIMPUS model SZX7 with a Galilean optical system at 0.8×–5.6× zoom range.

### 2.4. Sample Preparation

The biopolymer and different microparticles of wood were mixed mechanically varying the filler contents in increments of 10% from 0% to 30% (wt.%). Later, the different samples were extruded in a twin-screw co-rotating extruder, using the temperatures recommended by Solanyl^®^ C1201 (Zone 1: 135 °C–Zone 2: 150 °C–Zone 3: 160 °C–Die temperature: 165 °C) as a reference and at rotating speed of 60 rpm. Finally, a Meteor 270/75 injector supplied by Mateu-Sole^®^, (Barcelona, Spain) was used to obtain the samples for thermal and mechanical characterization.

### 2.5. Rheological Characterization

A Thermo Haake Rheoflixer MT1 capillary rheometer (Thermo Fisher Scientific, Karlsruhe, Germany) and current standard ISO 11443 was used to study the viscosity variations of all samples. The conditions of the tests were: temperature 160 °C, shearing speeds 100, 200, 500, 1000, 2000, 5000 and 10,000 s^−1^; diameter of die 1 mm, relation length/diameter 10, 20 and 30.

### 2.6. Mechanical Properties Measurement

Tensile and impact tests were used to evaluate the mechanical performance of Solanyl^®^ type reinforced with lignocellulose particle formulations. ELIB 30 electro-mechanical universal testing machine by Ibertest (S.A.E. Ibertest, Madrid, Spain) and Charpy impact machine (S.A.E. Ibertest, Madrid, Spain) were employed. UNE-EN-ISO 527 and ISO 179 standards were followed for tensile and impact tests, respectively. Five samples of each formulation were used in both tests and the mean and standard deviation of the measurements are reported.

### 2.7. Scanning Electron Microscopy (SEM) Measurements

The surface of the samples was metalized using gold sputter coating under vacuum conditions. Afterward, the different samples were analyzed in a ZEISS Ultra 55 Scanning Electronic Microscope (Carl Zeiss Iberia, Madrid, Spain).

### 2.8. Thermal Characterization

DSC Mettler-Toledo 821 equipment (Mettler-Toledo Inc., Schwerzenbach, Switzerland) allowed analysis of the different thermic transitions of all samples. The weight of the samples was 7–8 mg. A first heating (isothermal 5 min at 25 °C, and heating from 25 °C to 220 °C at 5 °C min^−1^) was completed, followed by a cooling process (from 220 °C to 30 °C at 5 °C min^−1^) and a second heating process (from 30 °C to 220 °C at 5 °C min^−1^). The tests were performed in a nitrogen environment (flow rate of 30 mL min^−1^).

The crystallinity of the PLA can be calculated using the following equation [32]:

(2) $$\chi_{c}=\frac{\Delta H_{m}-\Delta H_{cc}}{\Delta H_{m}^{0}}\times \frac{100}{w}$$

where:

$\chi_{c}$ is the degree of crystallinity in %
$\Delta H_{m}\;$is the melting enthalpy in J g^−1^
$\Delta H_{cc}\;$is the cold crystallization enthalpy in J g^−1^
$\Delta H_{m}^{0}$ is the calculated melting enthalpy of purely crystalline PLA, 93.7 J g^−1^
w is the weight fraction of the PLA sample.

Thermogravimetric Analysis, TGA, was carried out using a Mettler-Toledo TGA/SDTA 851 (Mettler-Toledo Inc., Schwerzenbach, Switzerland) with an initial temperature of 50 °C and final temperature of 800 °C, using a 20 °C min^−1^ heating rate. The analysis was carried out using a nitrogen atmosphere (20 mL min^−1^) in samples of 5 mg approximately.

To determine the Heat Deflection Temperature, a Vicat/HDT model Deflex 687-A2 (Metrotec, S.A, San Sebastian, Spain) was used. The oil used for the softening was silicon Dow Corning 200 Fluid 100 CS. The ISO 75 standard (B method) was followed employing a heating rate of 120 °C h^−1^ on samples sizing 80 × 10 × 4 mm^3^.

## 3. Results

### 3.1. Wood Flour Particles Characterization

**Figure 1:**
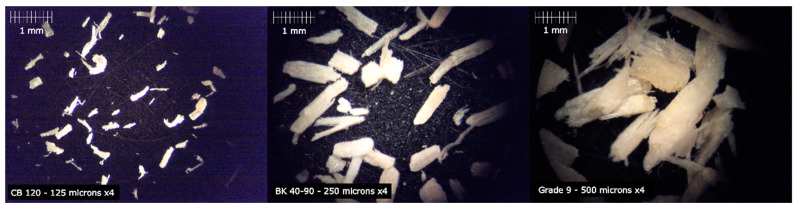
From left to right, micrographs of CB 120 retained on the sieve of 125 microns, BK 40-90 retained on the sieve of 250 microns and Grade 9 retained on the sieve of 500 microns.

The wood flour particles were characterized by optical microscopy, where it was possible to verify the presence of two types of particles, long and short, as shown in Figure 1. In the case of Lignocel^®^ CB120, fillers resulted in yellow particles with a fibrous structure. Meanwhile, Lignocel^®^ BK-40-90 and Lignocel^®^ Grade 9 resulted in long particles with cubic structure. This structure will further directly influence the processability as well as the final properties of Solanyl^®^ type-based composites.

**Figure 2:**
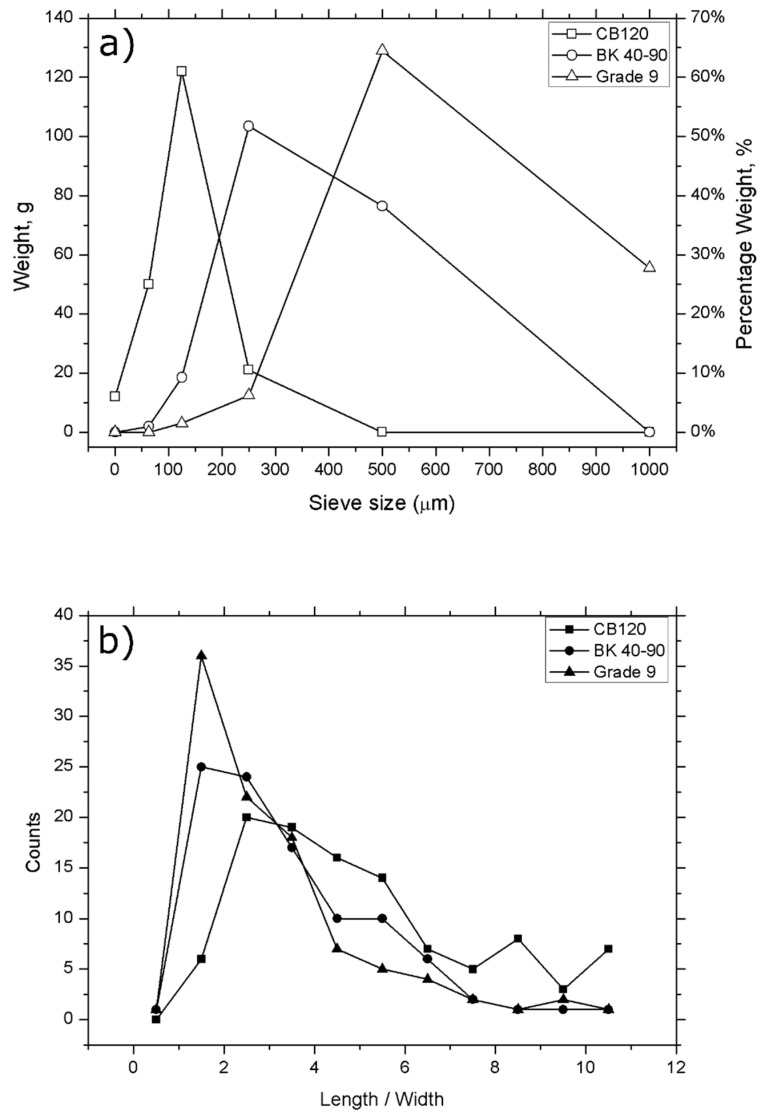
(**a**) Histogram of the weight distribution for different wood flour particles, and (**b**) histogram of the relationship between the length/width of different wood flour particles.

A granulometric study of all Lignocel^®^ particles with different sizes was carried out. The results are shown in Figure 2a, where it is possible to see the weight retained values in each of the sieves used. CB 120 sample possesses the smallest particles, and it is mostly composed of particles with sizes between 63 and 250 microns. Among the sizes, 125-micron particles are the most frequent. On the other hand, the BK 40-90 sample shows size particles between 250 and 1000 microns. Finally, 93% of particles of Grade 9 showed a size between 500 and 2000 microns, emphasizing 1000-micron particles, with 28%.

Mechanical properties of the composites depend on the size and particle distribution but are not the only factors, since the morphology of the particles is also very important. Since the optical micrographs showed significant differences in all wood flour particles (as shown in Figure 1), it was necessary to carry out a statistical study regarding the relationship between length and width (L/W) of the particles. The statistical analysis of different samples shows that the relation length/width decrease in bigger size samples and increases in smaller particles (CB 120), as shown in Figure 2b.

Another variable on which the mechanical properties of the composites material depend is the interaction of the phases (matrix phase (biopolymer) and disperse phase (wood flour)). Therefore, wood flour should be homogeneously dispersed among the Solanyl^®^ type bioplastic to obtain composites with improved properties. It is very important to have an optimal size distribution, and the Fuller and Thompson method is most often used due to its high simplicity. Table 2 shows the values obtained for the analysis of this methodology.

**Figure 3:**
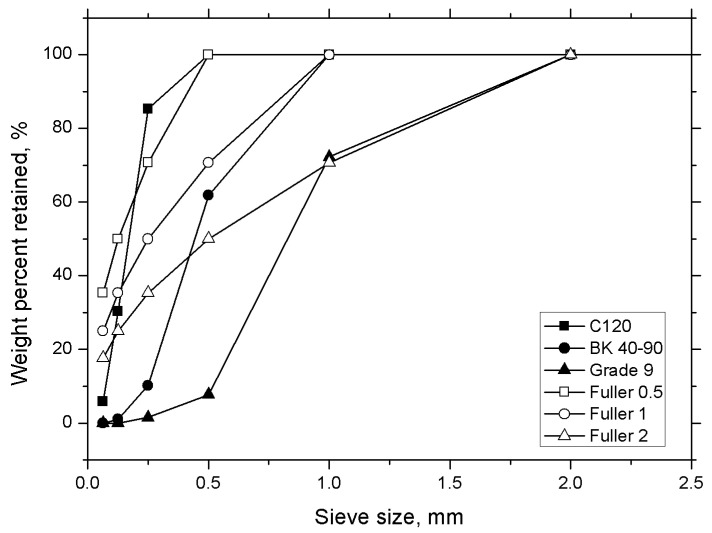
Representation of ideal particle size distribution (Fuller D = 0.5, D = 1, and D = 2) and wood flour size distribution.

From the obtained results, it is possible to establish that the D value for the samples is 0.5 mm for CB 120, 1 mm for BK 40-90 and 2 mm for Grade 9. The graphical representation of cumulative retained values versus sieve allowed observing differences between ideal particle size distribution and experimental values of wood flour (Figure 3). Finally, from the wood flour particles analysis, it can be concluded that the CB 120 sample is the most suitable particle for incorporation into the polymer matrix.

### 3.2. Biocomposites Characterization

Once the lignocellulosic particles were characterized, they were used to obtain biocomposites using Solanyl^®^ as the biopolymeric matrix, in 10, 20, and 30 wt.% loading. The Solanyl^®^/Lignocel-based composites were successfully processed by the melt-blending process followed by the injection molding process to simulate the industrial condition and, thus, assessing the possibility for their scalable production at the industrial level.

#### 3.2.1. Rheological Characterization

During the melt-extrusion and injection molding process, polymers and particularly biopolymers undergo degradation due to the strong shear stresses that act in the viscous molten polymer [33,34]. Thermal and mechanical characterization of material is extremely important, as this provides information on the variation produced by the introduction of filler, thermic treatments, ultraviolet radiation, and thermic cycles. However, if the material is going to be used at an industrial level, it is also important to establish the rheological characteristics to establish temperature and pressure parameters.

The use of capillary rheology requires the application of a range of corrections. There is a difference in the pressure between the radius of the deposit and the radius of the nozzle and the Bagley correction allows this value to be accurately obtained. The Rabinowitch correction allows the shear rate gradient to be corrected regarding the relationship between polymers and Newtonian fluids. After applying the Bagley and Rabinowitch corrections, the rheological curves show a slight increase in viscosity as the different wood particles are introduced (wood flour fillers). This behavior has been observed by other authors using polymers matrix. The viscosity of the PLA-based polymeric formulations increased with the addition of microparticles [35], while wood flour also showed the ability to increase the viscosity of polymeric matrix (i.e., polypropylene, PP) with increasing wood flour content. These studies showed that the interaction between polymer and particle produces an increase in viscosity of the end product material [36].

The same result can be observed in the evolution of values of viscosity in wood flour biocomposites, as shown in Table 3.

**Figure 4:**
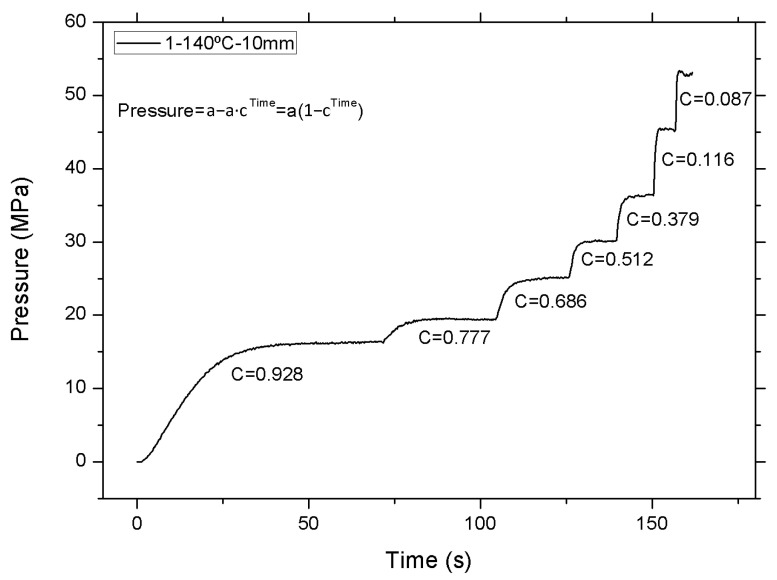
Evolution of the pressure versus time of the Solanyl^®^ at 140 °C with a nozzle length of 10 mm.

Increasing viscosity is not the only effect of adding wood flour to polymer matrices. When the behavior of the material subjected to high temperatures is analyzed in detail, the pressure shows a gradual increase as the shear rate increases from 100 to 10,000 s^−1^, as shown in Figure 4.

Rheological characterization is obtained from the constant pressure values for a given shear speed; subsequently, the application of various corrections (Bagley and Rabinowich) makes it possible to obtain the rheological curves of the material analyzed.

The variation in pressure versus time can be defined by the following equation:

(3) $$Pressure_{t}=P_{a}\left(1-C^{t}\right)$$

$P_{a\;}$is the pressure value when it has been stabilized for a particular shear rate in MPa.

C is the speed with which the pressure is achieved (dimensionless, always less than 1, with values close to zero suggesting that the rate at which the pressure increases is faster).

t is the time in s.

**Figure 5:**
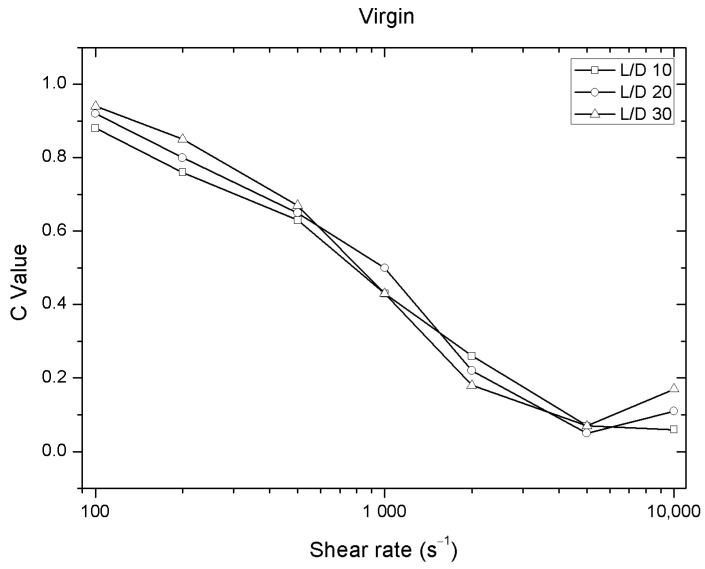
Evolution of C values versus shear rate for different nozzles length of the Solanyl^®^ at 140 °C.

The trend of C value is zero as the shear speed increases. This behavior can be observed in virgin material regardless of the type of nozzle used in the capillary rheometer. In this case, the values of C remain very close to each other for every shear speed, as shown in Figure 5.

**Figure 6:**
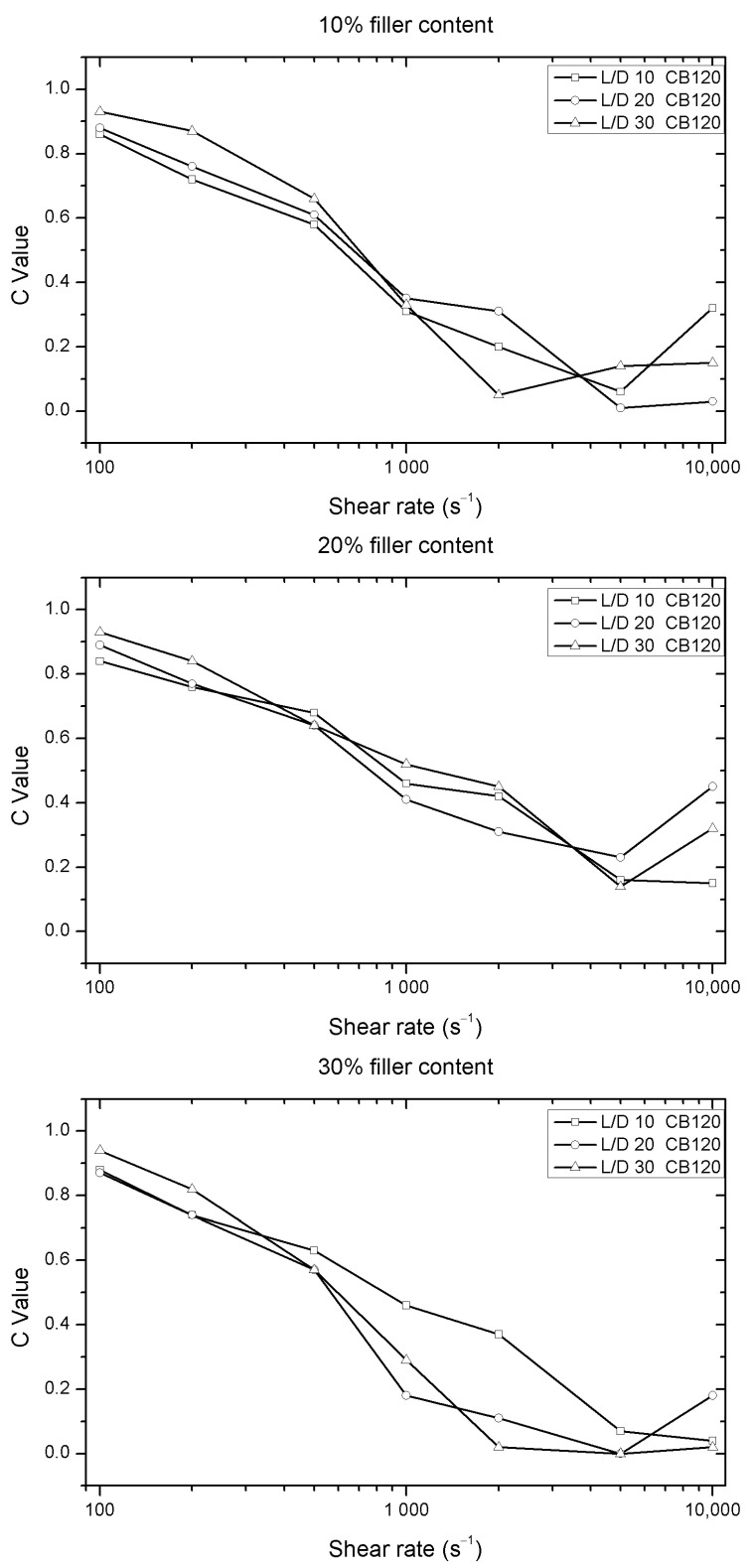
Evolution of C values versus shear rate for different nozzles length of the Solanyl^®^ with different percentages of fillers (CB 120).

The value of C is indicative of the rate at the pressure increases. This value can be affected by the presence of particles in the polymer matrix. C values of CB 120 and virgin polymer are very similar, but in the case of the smaller particle size (CB 120), the dispersion of C values increase when 2000 s^−1^ of shear rate is employed. This dispersion is greater in samples with 30 wt.% of wood flour and it is more evident in 1000 s^−1^ of shear rate, as can be seen in Figure 6.

**Figure 7:**
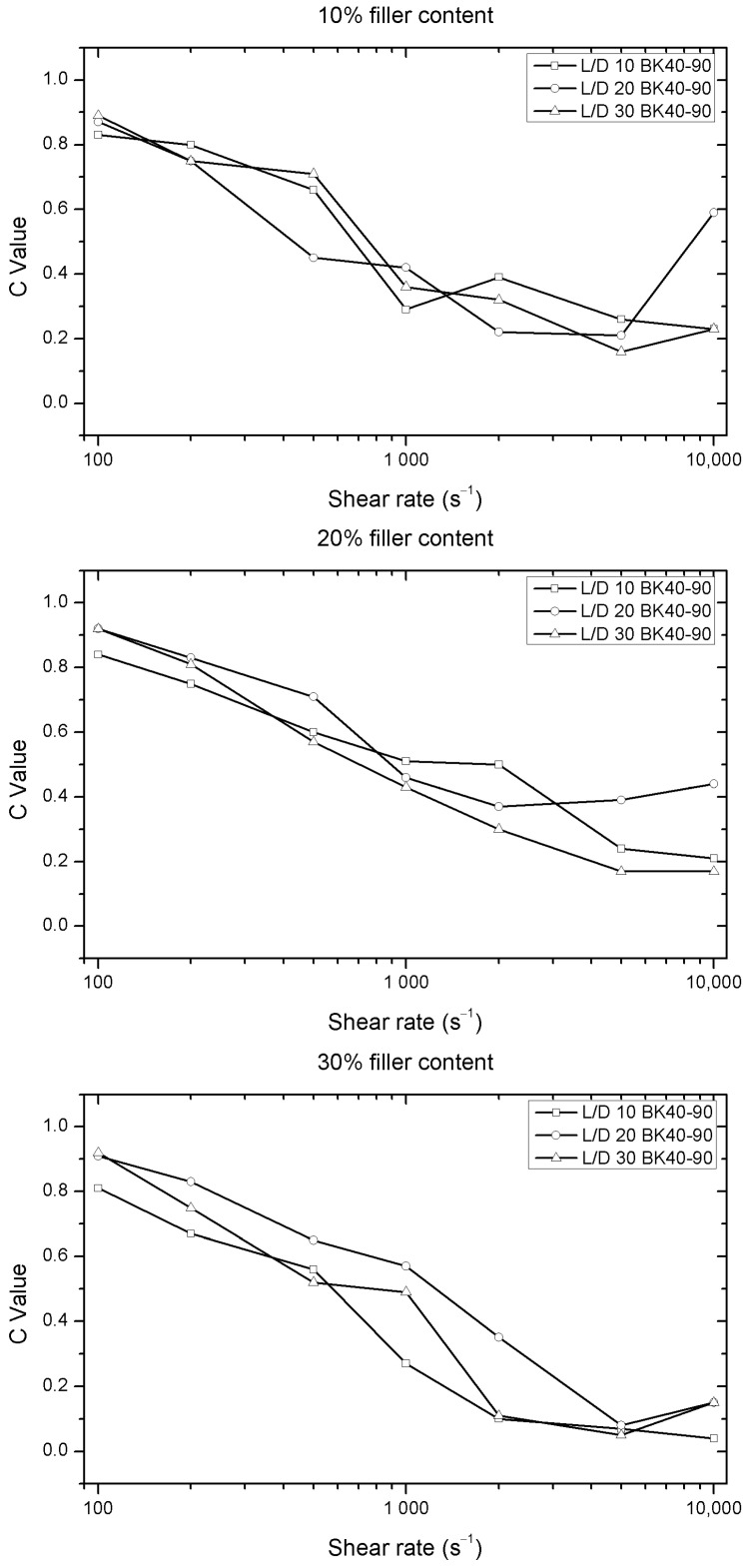
Evolution of C values versus shear rate for different nozzle lengths of the Solanyl^®^ with different percentages of fillers (BK 40-90).

On the other hand, BK 40-90 wood flour sample shows a behavior similar to CB 120. In this case, it is possible to see C value dispersion in the shear speed of 500 s^−1^, as seen in Figure 7. This behavior is due to the morphological properties of this sample, where 50% of the samples show lengths greater than 1 mm. Thus, the use of this sample can increase the probability of nozzle blockage.

**Figure 8:**
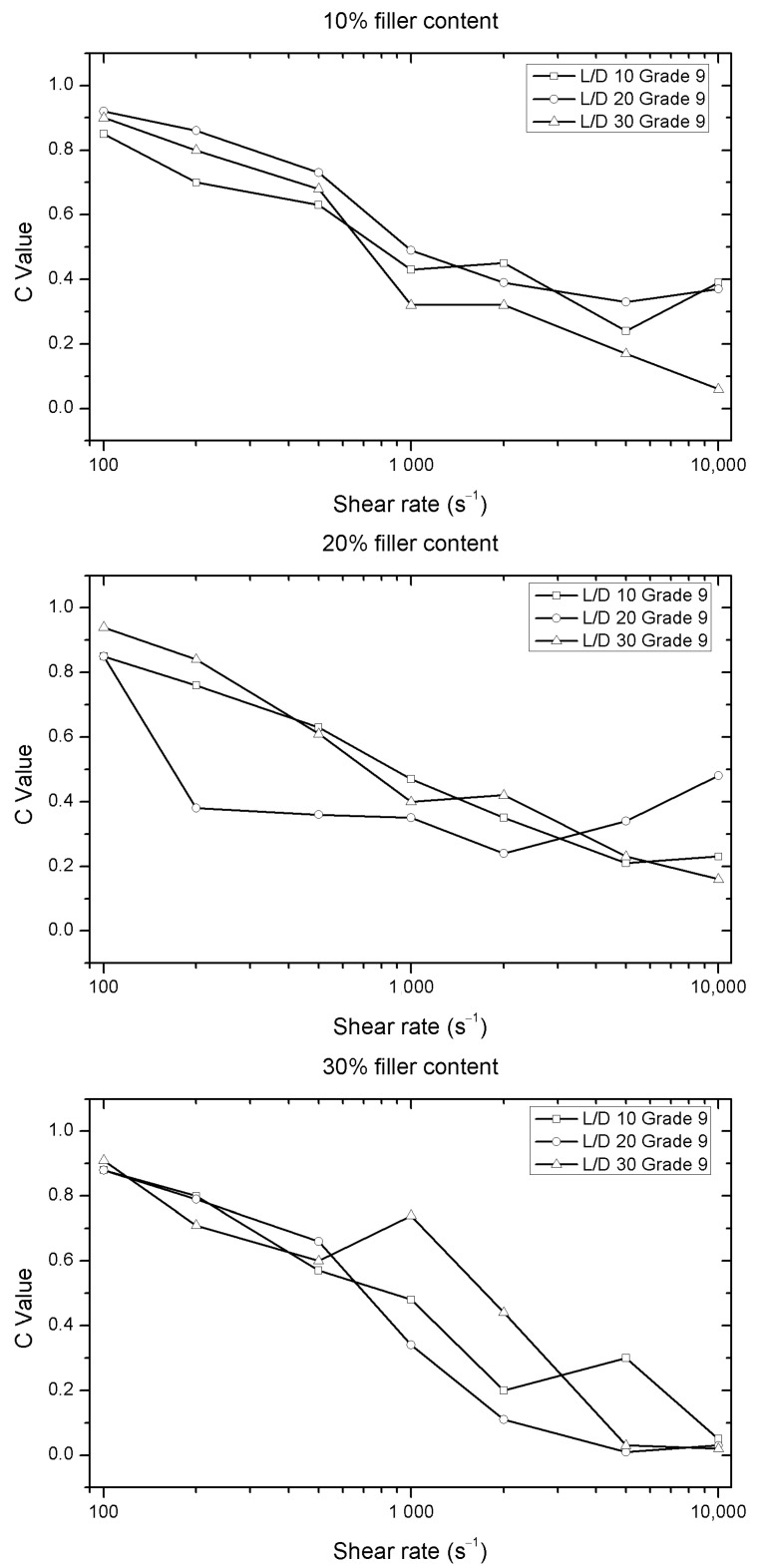
Evolution of C values versus shear rate for different nozzles length of the Solanyl^®^ with different percentages of fillers (Grade 9).

Finally, in the case of the largest particles (Grade 9), the dispersion of the C values is much greater in samples with wood flour contents of 20 and 30 wt.%. Moreover, this phenomenon appears at relatively low shear rates, as shown in Figure 8.

In summary, the presence of wood flour not only affects viscosity, but also means that the pressure increases much more quickly as greater percentages of filler are added. This behavior is repeated for all three types of particles used.

The variation in C values is attributed to the obstruction of the nozzle during the sample processing using the capillary rheometer. The fact that the pressure increases faster does not imply differences in rheological behavior, but it can cause certain problems in the manufacture of parts at an industrial level, especially in injection molding processes. The manufacture of parts by injection requires molds, which have sprues, runners, gates, and pieces. In sprues and runners, large enough sections are used to allow the material to flow without problems, whereas the reduction of the section in the case of gates is quite important and they are susceptible to obstruction by the presence of particles in the polymeric matrix.

#### 3.2.2. Mechanical Properties

Injection molded polymers for rigid applications (e.g., packaging, domestic appliance, toys, etc.) are required for high mechanical performance to overcome the strong shear stresses during processing in order to successfully obtain injected molded parts, as well as to offer good performance during service [33]. At the engineering level, the application of a material is often determined by its mechanical properties. Tensile strength, elongation at break, impact strength, and Young’s modulus should be analyzed.

Tensile strength is one of the most important properties; it is the characteristic that decides the maximum load that a material can support. Sometimes, the incorporation of filler may have a negative effect if there is no interaction between the matrix and the particle, as a consequence of non-homogeneous dispersion of the particle into the polymeric matrix. When the level of interaction is low, it can lead to a general decrease in mechanical properties. Nevertheless, when the interaction is good, due to the good dispersion of particles that reach a homogeneous distribution into the polymeric matrix, it allows an improvement of the overall mechanical performance.

**Figure 9:**
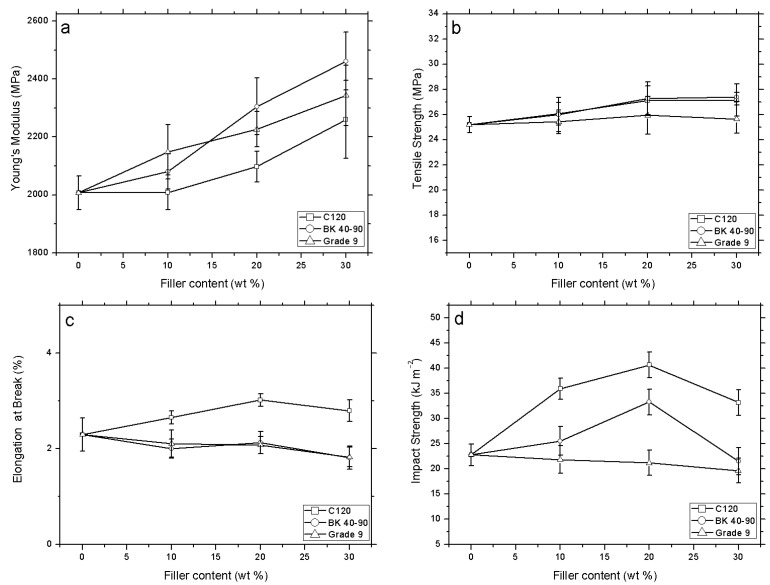
Mechanical properties of different samples: (**a**) Young’s modulus, (**b**) tensile strength, (**c**) elongation at break, (**d**) impact strength.

Young’s modulus of all biocomposites increased with the filler percentage, up to 20 wt.% (Figure 9a). The tensile strength showed a similar tendency when smaller fillers were used (Lignocel^®^ CB120 and BK 40-90) and they showed a slight improvement compared to the filler Grade 9, which has the largest sized particles. This last filler was unable to significantly increase the tensile strength of the Solanyl^®^ matrix (Figure 9b).

Regarding the elongation at break, for filler contents of 20 and 30 wt.%, a slight decrease is seen with Lignocel^®^ BK 40-90 and Lignocel^®^ Grade 9 particles. In contrast, the samples with smaller sized particles (Lignocel^®^ CB120) showed an increase in elongation at break up to 20% of wood flour content. However, these increasing tendencies slightly fall for samples with the highest content of 30 wt.% of this wood flour filler (Figure 9c).

Although the values for tensile strength and elongation at break were slightly improved in comparison with neat Solanyl^®^ type (polymeric matrix without filler), particularly with the smallest wood flour particles (Lignocel^®^ CB120), the application of high impact creates very different results for polymers with and without filler. In the samples with smaller sized particles (Lignocel^®^ CB120), the impact strength was practically the same in the formulations and the matrix (Figure 9d). Nevertheless, when the particle sizes are intermediate or large, the impact strength increases as the percentage of filler increases, achieving values up to 140% for Lignocel^®^ BK 40-90 and 180% for the biggest sized particles (Lignocel^®^ Grade 9). This maximum is produced in samples with a filler content of 20 wt.%. Once this content is surpassed, the matrix collapses and there is a huge decrease in the impact strength values. Other authors have observed increases in Young’s modulus depending on wood flour content. In contrast, the strain and Izod impact strength decreased [37].

#### 3.2.3. SEM Studies

The effect of the addition of wood flour particles with different granulometry on the microstructure of Solanyl^®^ type bioplastic was evaluated through SEM analysis, focusing on the dispersion of lignocellulose particles into the polymeric matrix, as well as on the interaction between wood flour particles with the polymeric matrix, to better understand the rheological and mechanical performance of the biocomposites. Considering the fibrous structure of lignocellulose fillers, the orientation into the polymeric matrix was also considered.

**Figure 10:**
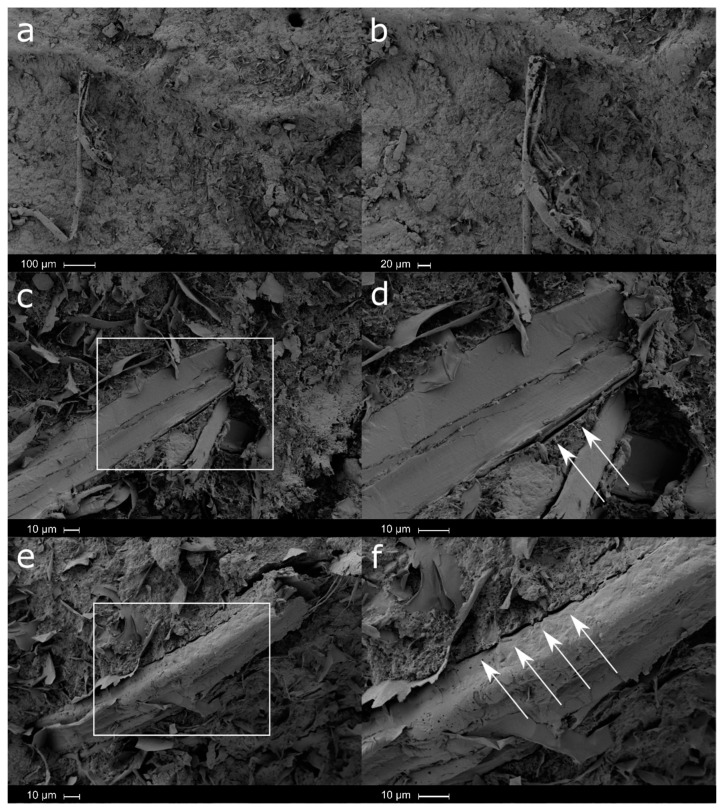
SEM micrographs of smallest particles, CB120—10 wt.% wood flour: (**a**) ×100, (**b**) ×200; CB120—20 wt.% wood flour: (**c**) ×500, (**d**) ×1000; and CB120—30 wt.% wood flour: (**e**) ×500, (**f**) ×1000.

In general, it is possible to observe oriented particles with a certain degree of inclination. Paying attention to the particle/matrix interphase, somewhat symptoms of lack of adhesion between the particle and the matrix phase are observed with the increasing amount of particle loading. Figure 10, shows the fracture surface of the produced Solanyl^®^/Lignocel biocomposites loaded with the smallest particles (CB 120, particles size between 63 and 250 microns) at different loadings of 10 wt.% (Figure 10a,b), 20 wt.% (Figure 10c,d) and 30 wt.% (Figure 10e,f). A clear homogeneous distribution of lignocellulose fillers in the polymeric matrix was observed, confirming the good processing ability of the smallest lignocellulose particles used here. Given their length and flexibility, it seems that CB 120 particles at 10 wt.% were well dispersed in the polymeric matrix, while non-oriented fibers were observed. Good adhesion between lignocellulose particles and the polymer matrix was observed, thus making the breaking process difficult. At higher loadings (20 wt.% Figure 10c,d) and particularly at 30 wt.% (Figure 10e,f), it seems that the interaction of the particles with the polymeric matrix is relatively low. Two phenomena can be observed depending on the orientation of the fibers. On one side, very thin fibers with a considerable length are partially attached to the polymeric matrix, while on the other side, small cavities are observed. This last phenomenon occurs when the orientation of the fiber is perpendicular to the working section.

**Figure 11:**
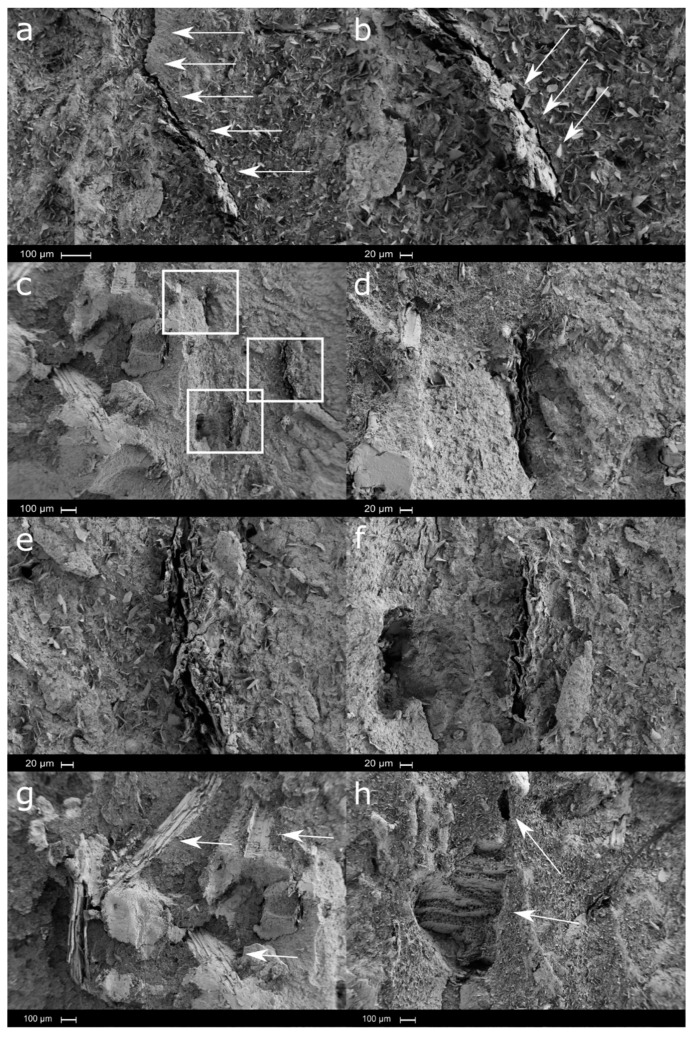
SEM micrographs of medium size particles—10 wt.% wood flour: (**a**) ×100, (**b**) ×200; 20 wt.% wood flour: (**c**) ×50, (**d**) ×200, (**e**) ×200, (**f**) ×200; 20 wt.% wood flour: (**g**) ×50; and 30 wt.% wood flour: (**h**) ×50.

Medium-sized particle (BK 40-90)-loaded biocomposites were characterized by partially maintaining the structure of the wood (Figure 11). In Figure 11a,b, the SEM images of Solanyl^®^ loaded with 10 wt.% of medium-sized particles (BK 40-90) are shown. Lignocellulosic particles appeared to be aligned with a certain degree of crushing, probably due to the wood grinding process, as well as due to the processing of biocomposites by melt-extrusion and injection molding. Although there is a homogeneous distribution of medium-sized particles, it can be observed that there are partially embedded lamellar particles into the polymeric matrix with evidence of fiber pull-out. Moreover, a clear crack is observed along the surface until it meets a particle, but borders it without great difficulty in fracturing.

Increasing the loading amount of medium-sized particles, lamellar particles tend to agglomerate, and the degree of crushing, produced either by the wood grinding process or by the shear stress suffered during biocomposite processing, became more evident (Figure 11c–f).

As occurred with the smallest particles, the medium-sized particles showed different orientations into the polymeric matrix, with more evidence of fiber pull-out, suggesting a lower degree of adhesion between the wood flour particles and the polymeric matrix (Figure 11g–h).

**Figure 12:**
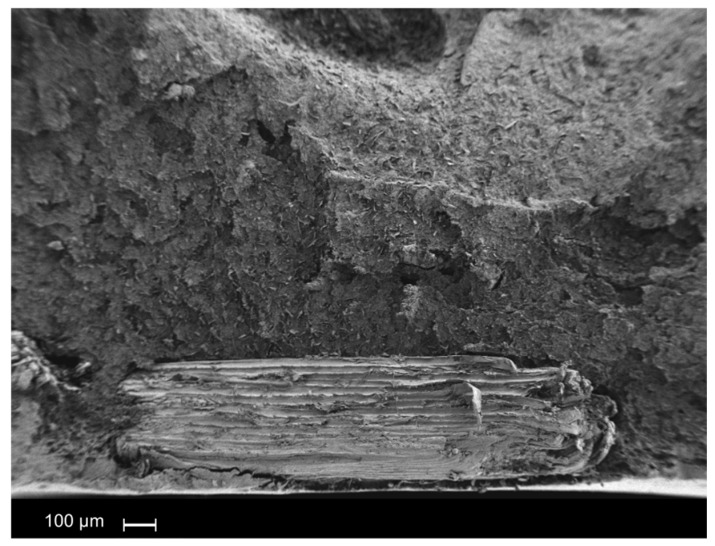
SEM micrographs of largest sized particles—10 wt.% of wood flour ×50.

Finally, the larger lignocellulose particles used present the lamellar structure of wood, with lengths of approximately 1 mm, and thus following the obtained weight distribution (Figure 12). Regarding the orientation of the fibers in the polymeric matrix, different orientations were found. Indeed, it is expected that large particles flow slowly in the polymer molten state, making their orientation difficult, as is evident from the near-surface region (Figure 12). Moreover, at the interface, fiber debonding, as well as fiber pull-out phenomena, are evident.

**Figure 13:**
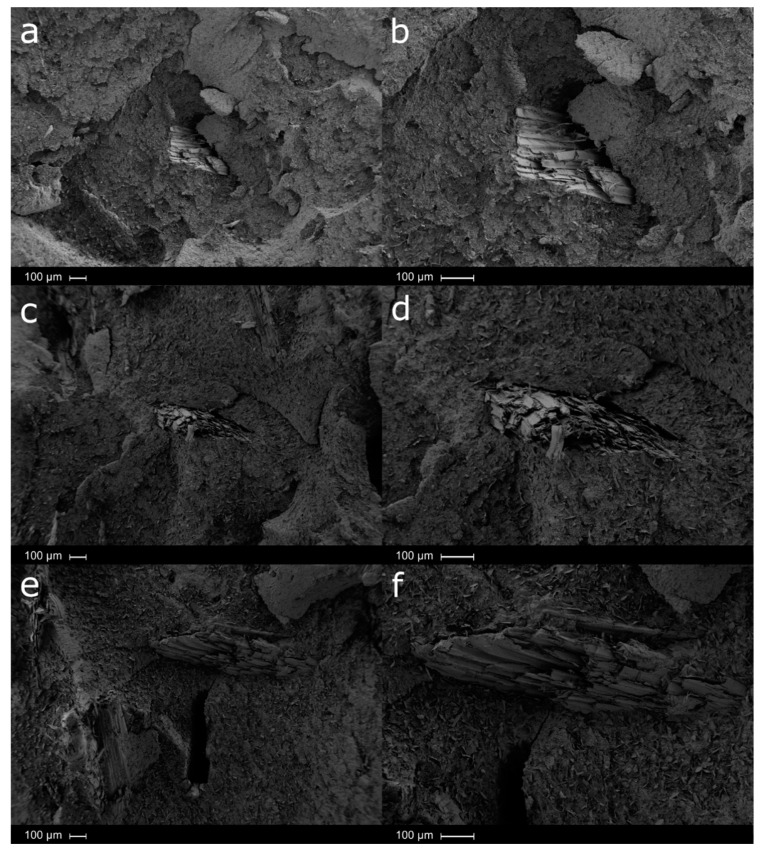
SEM micrographs of largest sized particles, Grade 9—10 wt.% wood flour: (**a**) ×50, (**b**) ×100; Grade 9—20 wt.% wood flour: (**c**) ×50, (**d**) ×100; and Grade 9—30 wt.% wood flour: (**e**) ×50, (**f**) ×100.

Instead, in the internal areas, it seems that the fibers acquire a certain degree of inclination. During the breaking process, the particle remains adhered to the polymeric matrix on one of its faces. Meanwhile, the other face is completely clear. This phenomenon was observed in all biocomposites prepared with the largest sized particles (Figure 13).

As a general conclusion, as the particle size increases, non-optimal adhesion and debonding phenomena are observed. A homogeneous and better dispersion was observed for the smallest particles used leading to the better mechanical response obtained.

#### 3.2.4. Thermal Properties

**Figure 14:**
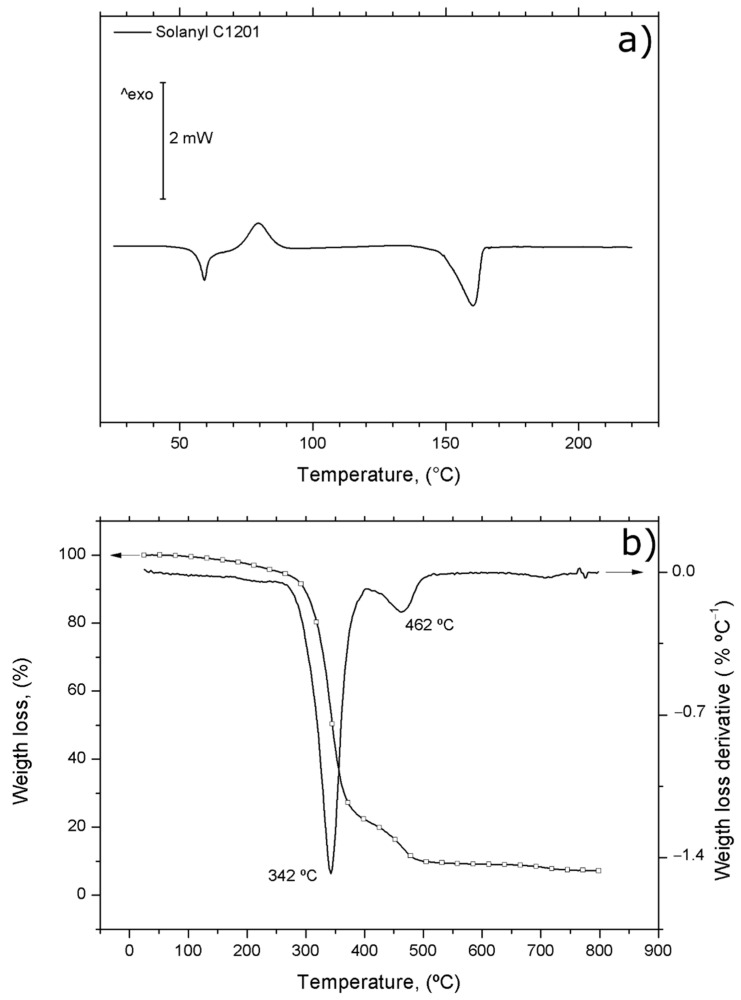
(**a**) DSC curve analysis of Solanyl^®^ C1201, (**b**) TGA and DTGA curves of Solanyl^®^ C1201.

Differential scanning calorimetry provides a range of information, such as glass transition temperature and crystallization temperature. Moreover, it allows mixtures of polymers to be detected. The DSC analysis (Figure 14a) shows three types of thermal curves and two endothermic reactions, one at around 60 °C, most probably due to the presence of PCL [38] in the compound, and another one at around 160 °C, which may correspond to the PLA phase (PLA) [11]. Furthermore, between 70 °C and 90 °C, an exothermic peak appears, which is related to the PLA cold crystallization. Moreover, the thermogravimetric curve (TGA) allows an understanding of the relationship between the matrix and dispersed phases. The maximum degradation rate of neat Solanyl^®^ C1201 is produced at 342 °C and 462 °C, corresponding to the thermal degradation of PLA and PCL phases, respectively (Figure 14b) [39]. The TGA analysis also revealed the presence of a starch-based material, as stated by the producer [8], since there is about a 5% mass loss at low temperatures (lower than 100 °C), as shown in Figure 14b [40].

**Figure 15:**
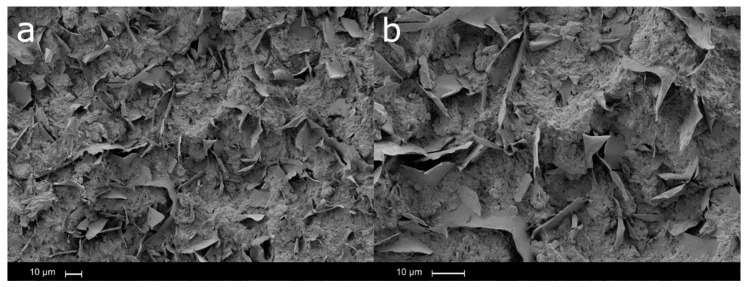
SEM micrograph of Solanyl^®^ C1201: (**a**) ×500 and (**b**) ×1000.

The presence of two phases in the neat polymeric matrix of Solanyl^®^ C1201 was further corroborated by SEM images which, at ×500, clearly shows the presence of both phases. This is relevant because the interaction between the two phases determines the mechanical properties of the material. The dispersed phase can be characterized as flakes embedded in the matrix phase (Figure 15) and the presence of the flakes confirm the presence of a starch-based material [41,42,43].

Understanding the matrix phases is extremely important when analyzing the behavior of a material, as are these phases that define the properties of the material. The dispersed phase, if it is homogeneously dispersed in the polymeric matrix, will further improve the overall performance of the final material. It can be seen that the first rise is much greater than the second, indicating that the matrix phase is composed of a higher amount of PLA (around 80%) and around 20% of PCL (Figure 14b). This data are again extremely important, as the mechanical properties are governed by the interaction between the PLA and the PCL matrices, as well as between both polymeric matrices and the lignocellulosic additive particles. Furthermore, the Solanyl^®^ C1201 biopolymer has a degree of crystallinity (as shown in DSC results in Figure 14a). This is significant, as crystallinity is often an important factor in deciding the mechanical behavior of a polymer. Any variation in this characteristic will have repercussions on the mechanical properties of the final material. Wood flour was added to increase the crystallinity of the Solanyl^®^ type bioplastic.

Table 4 shows the DSC thermal parameters and the evolution of crystallinity in the Solanyl^®^ type biocomposites reinforced with Lignocel^®^ particles from both the first and second heating DSC scans. In the first heating scan, the cold crystallization exothermic peak was observed in all formulations, showing that the injection molding processing conditions did not produce the crystallization of PLA, and thus it crystallizes during DSC heating. The degree of crystallinity in general increases with an increasing amount of lignocellulosic particles, particularly in small (Lignocel^®^ CB 120) and middle-sized particles (Lignocel^®^ BK 40-90), showing the ability of wood flour to act as a nucleating agent. However, the crystallinity from the first heating cannot be taken as representative as the cooling conditions varied from sample to sample. To eliminate the effect of the cooling rate during processing, and thus the thermal history, a second DSC heating was carried out. The DSC cooling conditions applied made it possible to crystallize PLA, and thus it did not crystallize during the second heating scan. In this case, the crystallinity values do indeed better represent the effect of the presence of lignocellulose particles in the samples.

It is worth highlighting the fact that crystallinity increases depending on the presence of lignocellulosic filler regardless of the particle size, although the increase is very slight for samples with the larger sized particles, Lignocel^®^ Grade 9. This trend in the crystallinity values was observed by other authors in composites based on PLA with pinewood flour composites [44,45].

In both cases, crystallinity increases depending on the quantity of filler present. Moreover, the use of blends in polymers led to significant advances in this field, including the creation of materials with properties that the individual materials do not have. The difficulty, however, is that on certain occasions, thermal transitions are hidden by the presence of another polymer. This is the case with the glass transition temperature (T_g_) of PLA, which is concealed by the melting of PCL. The difficulty to determine the T_g_ during the DSC heating scans due to its overlapping with the melting peak of PCL frequently occurs in PLA/PCL-based formulations [12,13,14].

Finally, the heat deflection temperature (HDT) values remain virtually unchanged regardless of the type and percentage of filler used, with a slight increase only in the samples with 30 wt.% filler, as shown in Table 5. This indicates good interaction between polymer and filler [46,47].

## 4. Conclusions

The present study focused on the influence of three types of wood flour, Lignocel^®^ C120 (70–150 µm), BK 40-90 (300–500 µm) and Grade 9 (800–1100 µm), on the rheological, mechanical, and thermal properties of Solanyl^®^ type bioplastic. The main novelty of this study is the analysis of the pressure variation during the rheological study of the prepared samples. The granulometric and morphological study of different wood flour samples and the application of the Fuller-Thompson method allowed us to determine which sample presented better characteristics for improving the properties of the polymeric mixture (polymer—wood flour) to obtain biocomposites with improved performance. The rheological study revealed an increase in viscosity as the filler percentage increases. The viscosity data are especially relevant for use at the industrial level, particularly by extrusion and injection molding processes. Despite the low viscosity variation as the percentage of filler increased, a more detailed analysis showed that pressures rose more quickly, which can be attributed to the obstruction of the nozzle by the presence of wood flour particles. This fact could cause an increase in defective pieces due to the obstruction of the gate inside the mold. All these results indicate that the material can be processed by extrusion.

The tensile test results showed that, in general, Young’s modulus gave increasing values for all types of particles. Similarly, the tensile strength increased with an increasing amount of wood flour small-sized (CB 120) and medium-sized particles (BK 40-90) in the formulation. The largest particles (Grade 9) did not produce significant changes in the tensile strength values. The elongation at break showed little variation with the addition of different percentages of medium-sized particles (BK 40-90) and the largest particles (Grade 9) to the Solanyl^®^ type bioplastic. Nevertheless, the incorporation of the smallest lignocellulosic microparticles (CB 120) increased the elongation at break. The impact strength was only affected by the incorporation of small and medium-sized particles (CB 120 and BK 40-90) showing a maximum value for biocomposites with 20 wt.% wood flour filler. SEM images revealed good interaction between particles and the Solanyl^®^ type polymeric matrix, which positively affects the mechanical behavior of the biocomposites. On the other hand, an increase in crystallinity leads to an increase in the mechanical properties, visible through the impact strength and Young’s modulus. Of all the samples analyzed, those with 10 or 20 wt.% filler of the smallest particles (CB120) and medium-sized particles (BK 40-90) showed the best combination for processing by injection molding as well as leading to biocomposites with improved properties and, thus, that are of interest for sustainable industrial applications.

## Figures and Tables

**Table 1:** Physical properties of Lignocel^®^ fillers.

	CB120	BK 40-90	Grade 9
Color	Yellow	yellow	yellow
Structure	Fibrous	cubic	cubic
Particle range (mm)	0.07–0.15	0.30–0.50	0.80–1.10

**Table 2:** Weight values retained (g), cumulative retained (%), and passed of different particles CB 120, BK 40-90 and Grade 9.

	CB 120				BK 40-90				Grade 9			
Mesh Size	Weight Retained	Cumulative Retained		Total % Passing	Weight Retained	Cumulative Retained		Total % Passing	Weight Retained	Cumulative Retained		Total % Passing
mm	g	g	%	%	g	g	%	%	G	g	%	%
32	0	0	0	100	0	0	0	100	0	0	0	100
16	0	0	0	100	0	0	0	100	0	0	0	100
8	0	0	0	100	0	0	0	100	0	0	0	100
4	0	0	0	100	0	0	0	100	0	0	0	100
2	0	0	0	100	0	0	0	100	0	0	0	100
1	0	0	0	100	0	0	0	100	55.5	55.5	27.75	72.25
0.5	0	0	0	100	76.5	76.5	38.15	61.85	129	184.5	92.25	7.75
0.25	31	31	15.12	84.88	103.5	180	89.77	10.23	12.5	197	98.5	1.5
0.125	112	143	69.75	30.25	18.5	198.5	99.00	1	3	200	100	0
0.063	50	193	94.14	5.86	2	200.5	100	0	0	200	100	0
bottom	12	205	100	0	0	200.5	100	0	0	200	100	0
Total	205				200.5				200			

**Table 3:** Viscosity values for Solanyl^®^ composites with a varying wood flour fraction, C120, BK 40-90, and Grade 9.

		Viscosity, Pa s						
Shear rate (s^−1^)		100	200	500	1000	2000	5000	10,000
Material	% of filler							
Virgin	0	3061.25	1730.12	815.25	463.87	236.75	90.38	49.92
CB 120	10	3244.37	1900.46	842.38	500.87	250.78	95.31	52.85
	20	3300.25	2000.65	974.27	520.51	251.09	95.91	59.22
	30	3492.52	2101.56	985.37	543.18	280.06	103.15	63.23
BK 40-90	10	3408.25	2109.37	1022.62	532.46	256.84	110.56	60.87
	20	3662.51	2271.56	1095.25	587.18	295.46	120.87	70.23
	30	3720.21	2500.32	1200.54	610.87	306.43	126.32	75.98
Grade 9	10	3304.37	2036.87	1021.62	565.87	291.90	110.28	60.67
	20	3400.25	2286.56	1109.62	598.56	309.81	118.95	65.99
	30	3563.12	2500.65	1200.87	620.15	325.46	120.38	70.15

**Table 4:** Enthalpy values of different samples and the calculated crystallinity.

			First Heating at 5 °C min^−1^		
Mixture	Tcc, °C	Tm, °C	Normalized Enthalpy		Crystallinity $\chi_{c}$, (%)
			Cold (J g^−1^)	Hot (J g^−1^)	
PLA-PCL	78.85	160.17	6.43	14.31	10.51
10—CB 120	78.77	161.86	6.40	14.70	12.31
20—CB 120	79.72	161.91	4.70	15.43	17.90
30—CB 120	79.83	160.58	4.42	16.80	23.61
10—BK 40-90	79.94	161.28	3.60	12.15	12.67
20—BK 40-90	80.35	163.03	5.84	14.18	13.90
30—BK 40-90	80.36	162.74	5.17	14.85	18.45
10—Grade 9	82.50	161.64	5.92	13.27	10.89
20—Grade 9	79.77	162.23	4.77	13.61	14.73
30—Grade 9	79.39	160.28	4.23	12.56	15.88
	**Second Heating at 5 °C min^−1^**				
**Mixture**	**Tcc, °C**	**Tm, °C**	**Normalized** **Cold** **(J g^−1^)**	**Enthalpy** **Hot** **(J g^−1^)**	**Crystallinity** $\chi_{c}$ **, (%)**
PLA-PCL	-	156.86	-	12.62	16.84
10—CB 120	-	158.21	-	12.17	18.04
20—CB 120	-	158.19	-	12.62	21.05
30—CB 120	-	158.35	-	14.11	26.89
10—BK 40-90	-	157.71	-	12.43	18.43
20—BK 40-90	-	159.88	-	13.66	22.78
30—BK 40-90	-	159.42	-	14.28	27.22
10—Grade 9	-	158.16	-	10.66	15.80
20—Grade 9	-	158.58	-	12.16	20.28
30—Grade 9	-	157.23	-	11.12	21.19

**Table 5:** HDT values (°C) of different samples with wood flour.

Filler Content	CB 120	BK 40-90	Grade 9
wt.%			
0	53.8	53.8	53.8
10	53.8	54.2	54.4
20	54.4	54.6	54.8
30	54.8	55.0	55.3

## Data Availability

All relevant data (mechanical properties, SEM micrographs, Thermal Analysis curves) are available via e-mail (fraparga@dimm.upv.es).
